# On Physical Properties of Tetraether Lipid Membranes: Effects of Cyclopentane Rings

**DOI:** 10.1155/2012/138439

**Published:** 2012-09-18

**Authors:** Parkson Lee-Gau Chong, Umme Ayesa, Varsha Prakash Daswani, Ellah Chay Hur

**Affiliations:** Department of Biochemistry, Temple University School of Medicine, 3420 North Broad Street, Philadelphia, PA 19140, USA

## Abstract

This paper reviews the recent findings related to the physical properties of tetraether lipid membranes, with special attention to the effects of the number, position, and configuration of cyclopentane rings on membrane properties. We discuss the findings obtained from liposomes and monolayers, composed of naturally occurring archaeal tetraether lipids and synthetic tetraethers as well as the results from computer simulations. It appears that the number, position, and stereochemistry of cyclopentane rings in the dibiphytanyl chains of tetraether lipids have significant influence on packing tightness, lipid conformation, membrane thickness and organization, and headgroup hydration/orientation.

## 1. Introduction

Archaea are subdivided into two kingdoms: euryarchaeota and crenarchaeota [1]. Euryarchaeota include methanogens and halophiles, whereas crenarchaeota are traditionally comprised of thermophilic or hyperthermophilic archaea [1]. Halophiles and some methanogens are found mostly in high salt water or hypersaline systems such as natural brines, alkaline salt lakes, and salt rocks; while thermophilic and hyperthermophilic archaea are found in very high temperature environments [2]. In recent years, crenarchaeota have also been found in nonextreme environments such as soil and pelagic areas [3, 4].

**Figure 1:**
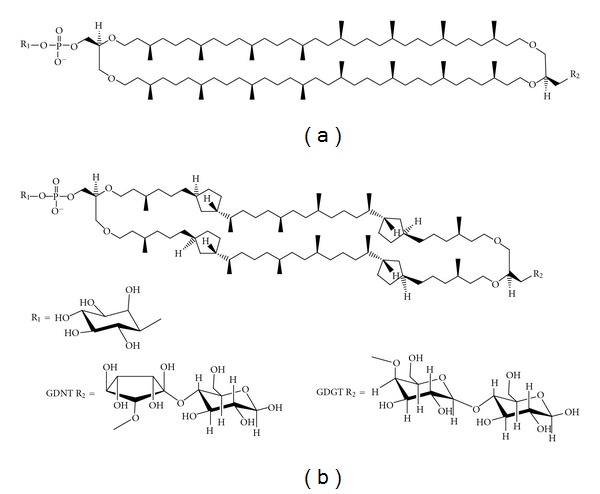
Illustrations of the molecular structures of the bipolar tetraether lipids in the polar lipid fraction E (PLFE) isolated from *S. acidocaldarius*. PLFE contains (a) GDGT (or caldarchaeol) and (b) GDNT (or calditolglycerocaldarchaeol). The number of cyclopentane rings in each biphytanyl chain can vary from 0 to 4. The different head groups of GDNT and GDGT are presented at the bottom. GDG(N)T-0 and GDG(N)T-4 contain 0 and 4 cyclopentane rings per molecule, respectively (taken from [5], reproduced with permission).

The plasma membranes of archaea are rich in tetraether lipids (TLs) and diphytanylglycerol diethers, also known as archaeols (reviewed in [11–13]). TLs are the dominating lipid species in crenarchaeota, particularly in thermoacidophilic archaea (~90–95%). They are also found in methanogens (0–50%) but are virtually absent in halophiles. Archaeal TLs contain either a caldarchaeol (GDGT) or a calditoglycerocaldarchaeol (GDNT) hydrophobic core (Figure 1) [13–17]. GDGT has two glycerols at both ends of the hydrophobic core. GDNT has a glycerol backbone at one end of the hydrophobic core and the calditol group at the other end. Typically, TLs in methanogens contain only GDGT, but TLs in thermoacidophiles, particularly in the members of the order *Sulfolobales*, have both GDGT and GDNT components. The *Metallosphaera sedula TA-2* strain from hot springs in Japan, which has only GDGT-based lipids, is an exception [18]. TLs have been thought to play an important role in the thermoacidophile's high stability against extreme growth conditions such as high temperatures (e.g., 65-90°C) and acidic environments (e.g., pH 2-3) [19]. However, more recent studies showed that GDGT-based TLs are also abundant in nonextremophilic crenarchaeota present in marine environments, lakes, soils, peat bogs, and low temperature areas [20, 21]. The functional role of tetraether lipids in crenarchaeota is not fully understood.

The hydrophobic core of archaeal TLs is made of dibiphytanyl hydrocarbon chains, which may contain up to 8 cyclopentane rings per molecule (reviewed in [13]). The number of cyclopentane rings increases as growth temperature increases [22–25], but decreases with decreasing pH in growth media [26]. The presence of cyclopentane rings is a structural feature unique for archaeal tetraether lipids. Therefore, it is of great interest to unravel its biological roles.

Various polar headgroups can be attached to the glycerol and calditol backbones and yield either monopolar or bipolar tetraether (BTL) lipids. Archaeal BTLs are glycolipids or phosphoglycolipids (illustrated in Figure 1). Liposomes that are made of BTLs containing two or more sugar moieties exhibit lower proton permeability than those containing only one sugar molecule [26]. It has been proposed that thermoacidophilic archaea cells adapt to low pH and high temperature by increasing the number of sugar moieties and cyclopentane rings [26, 27]. Increasing the number of cyclopentane rings tightens membrane packing (discussed later) [27]. Sugar moieties and the phosphate group in the BTL polar headgroup regions interact with each other to form a strong hydrogen bond network at the membrane surface [28].

BTLs are unique to archaea and cannot be biosynthesized by eukaryotic or bacterial cells. The ether formation from glycerol has been studied to a great extent ([29] and references cited therein). The calditol moiety of GDNTs can be synthesized via an aldol condensation between dihydroxyacetone and fructose [30]. Calditol is then reduced and alkylated to form GDNTs [30]. An *in vitro* study showed that with the aid of 1L-*myo*-inositol 1-phosphate synthase, archaetidylinositol phosphate (AIP) synthase and AIP-phosphatase, archaeal inositol phospholipid (see Figure 1 e.g.) can be formed from CDP-archaeol and D-glucose-6-phosphate via *myo-*inositol-1-phosphate and AIP [31]. It has been proposed that the cyclopentane rings in BTLs of *Sulfolobus* are synthesized from glucose by a “cyclase” enzyme of the calditol carbocycle [32].

In this paper we focus on the recent findings related to the physical properties of tetraether lipid membranes, with special attention on the effects of the number, position, and configuration of cyclopentane rings on membrane properties. We discuss the findings obtained from model membranes composed of naturally occurring archaeal tetraether lipids and synthetic tetraethers as well as the results from computer simulations.

## 2. Physical Properties of Model Membranes Composed of Thermoacidophilic Tetraether Lipids

### 2.1. Membranes Made of Total Polar Lipid Extracts

The stability and physical properties of liposomes made from the total polar lipids (TPLs) extracted from archaea have been studied extensively (reviewed in [11, 12, 33, 34]). TPL extracts contain both diether and tetraether lipids. The general trend shows that membranes become more stable as the mole fraction of tetraether lipids increases. As an example, liposomes made of diether lipids such as *Methanosarcina mazei *TPL (0 wt% in caldarchaeols) were unstable against simulated human bile while those made of TPL from *Methanobacterium espanolae* (65% in caldarchaeols) and *Thermoplasma acidophilum* (90% in caldarchaeols) were relatively more stable [35]. Solute and water permeability also decrease as the content of tetraether lipids in membranes made with archaeal TPLs increases [36].

Sprott et al. [37] demonstrated that liposomes made with TPL from the archaeon *M. smithii AL1* can be highly fusogenic when exposed to low pH and *α*- and *β*-glucosidases. It was suggested that, at low pH (4.8), the positively charged glucosidases interact with the anionic phospholipids in *M. smithii *TPL, which in turn causes archaeosomes to rapidly aggregate [37]. Aggregation is a prerequisite for membrane fusion. This result is somewhat surprising because previous studies showed that tetraether liposomes are resistant to fusogenic compounds [38–40]. Since TPL of *M. smithii AL1* contains a significant amount of diethers, in addition to caldarchaeols (~40 wt %), it is possible that the strong fusogenic activity mentioned above comes from the diether component.

### 2.2. Membranes Made of Partially Purified Tetraether Lipid Fractions

Since tetraethers are the dominating lipid species in thermoacidophiles, and the presence of diethers in the total polar lipid extracts makes the data interpretation more difficult, it is of biophysical interest to study membranes made only with tetraether lipids. The physical properties of lipid membranes made of partially purified polar lipid fractions from the archaeon *Sulfolobus solfataricus* have been reviewed [11, 34]. In this section, we focus on the recent studies of membranes made of partially purified polar lipid fractions isolated from the archaeon *Sulfolobus acidocaldarius*.

#### 2.2.1. PLFE

The polar lipid fraction E (PLFE) is one of the major bipolar tetraether lipids (BTLs) found in the thermoacidophilic archaeon *S. acidocaldarius *[41, 42]. PLFE is a mixture of GDNT and GDGT (Figure 1). The GDNT component (~90% of total PLFE) contains phospho-*myo*-inositol on the glycerol end and *β*-glucose on the calditol end, whereas the GDGT component (~10% of total PLFE) has phospho-*myo*-inositol attached to one glycerol and *β*-D-galactosyl-D-glucose to the other glycerol skeleton (Figure 1). The nonpolar regions of these lipids consist of a pair of 40-carbon biphytanyl chains, each of which may contain up to four cyclopentane rings [22].

#### 2.2.2. PLFE Liposomes

PLFE lipids can form stable unilamellar (~60–800 nm in diameter), multilamellar, and giant unilamellar (~10–150 *μ*m) vesicles [40, 41, 43]. The lipids in these vesicles span the entire lamellar structure, forming a monomolecular thick membrane [44], which contrasts to the bilayer structure formed by monopolar diester (or diether) phospholipids. Compared to liposomes made of diester or diether lipids, PLFE liposomes exhibit extraordinary membrane properties (reviewed in [11, 12, 34]). PLFE liposomes exhibit low proton permeability and dye leakage [45, 46], high stability against autoclaving and Ca^2+^-induced vesicle fusion [40, 47], tight and rigid membrane packing [43], and low enthalpy and volume changes associated with the phase transitions [48, 49].

It is known that a decrease in archaeal cell growth temperature (*T*_*g*_) decreases the number of cyclopentane rings in archaeal TLs [22]. In the case of *S. acidocaldarius*, the average number of cyclopentane rings per tetraether lipid molecule decreases from 4.8 to 3.4 when *T*_*g*_ drops from 82°C to 65°C [23]. Recent experimental work (see below) has addressed the effect of *T*_*g*_, inferentially the number of cyclopentane rings, on the physical properties of tetraether lipid membranes.

#### 2.2.3. Effect of Cyclopentane Rings on Phase Behavior of PLFE Liposomes

The phase behavior of PLFE liposomes has been characterized by small angle X-ray scattering, infrared and fluorescence spectroscopy, and differential scanning calorimetry (DSC). PLFE liposomes exhibit two thermally-induced lamellar-to-lamellar phase transitions at ~47–50°C and ~60°C [34, 43, 48, 49] and a lamellar-to-cubic phase transition at ~74–78°C [48, 49] all of which involve small or no volume changes as revealed by pressure perturbation calorimetry (PPC) [49]. The calorimetry experiments also suggested that the number of cyclopentane rings in the dibiphytanyl chains affect membrane packing in PLFE liposomes because the liposomes derived from different cell growth temperatures showed different thermodynamic properties [49]. DSC allows us to determine the enthalpy change (Δ*H*) of the phase transition. PPC, on the other hand, allows us to determine the relative volume change (Δ*V*/*V*) at the phase transition and the thermal expansivity coefficient (*α*) at each temperature.

For PLFE liposomes derived from cells grown at 78°C, the DSC heating scan exhibited an endothermic transition at 46.7°C, which can be attributed to a lamellar-to-lamellar phase transition and has an unusually low Δ*H* (3.5 kJ/mol), when compared to that for the main phase transitions of saturated diacyl monopolar diester lipids (e.g., 1,2-dimyristoyl-*sn*-glycero-3-phosphocholine, DMPC). The PPC scan revealed that, at this same phase transition, the relative volume change (Δ*V*/*V*) in the membrane is very small (~0.1%) and much lower than the Δ*V*/*V* value 2.8% for the main phase transition of DMPC. The low Δ*H* and Δ*V*/*V* values may arise from the restricted *transauche* conformational changes in the dibiphytanyl chain due to the presence of cyclopentane rings, branched methyl groups, and to the spanning of the lipid molecules over the whole membrane [49].

For PLFE liposomes derived from cells with growth temperature of 65°C, similar DSC and PPC profiles were obtained. However, the lower cell growth temperature yielded a higher Δ*V*/*V* (~0.25%) and Δ*H* (14 kJ/mol) value for the lamellar-to-lamellar phase transition measured at pH 2.1. The lower growth temperature also generated less negative temperature dependence of *α*. The changes in Δ*V*/*V*, Δ*H*, and the temperature dependence of *α* can be attributed to the decrease in the number of cyclopentane rings in PLFE due to the lower growth temperature [49]. A decrease in the number of cyclopentane rings makes the membrane less tight and less rigid; thus, a higher Δ*V*/*V* value is shown through the phase transition.

#### 2.2.4. Effect of Cyclopentane Rings on Compressibility and Membrane Volume Fluctuations of PLFE Liposomes

The isothermal and adiabatic compressibility and relative volume fluctuations of PLFE liposomes have been determined by using calorimetry (DSC and PPC) and molecular acoustics (ultrasound velocimetry and densimetry) [50]. The compressibility values of PLFE liposomes were low, compared to those found in a gel state of 1,2-dipalmitoyl-*sn*-glycero-3-phosphocholine (DPPC) [50]. Relative volume fluctuations of PLFE liposomes at any given temperature examined were 1.6–2.2 times more damped than those found in DPPC liposomes [50]. Volume fluctuations are closely related to solute permeation across lipid membranes [51] and lateral motion of membrane components [52]. Thus, the low values of relative volume fluctuations explain why PLFE liposomes exhibit unusually low proton permeation and dye leakage [45, 46] as well as limited lateral mobility, especially at low temperatures (e.g., <26°C) [43, 53].

**Figure 2:**
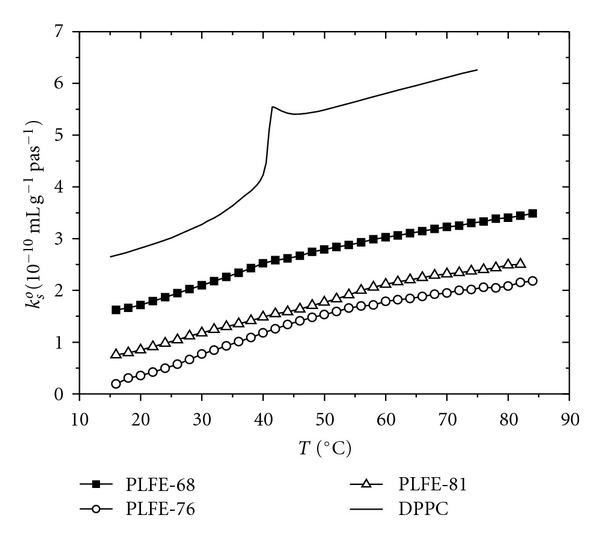
Adiabatic compressibilities (*k*_*S*_^*o*^) of PLFE liposomes derived from cells grown at three different temperatures: 68°C (dark squares), 76°C (open circles), and 81°C (open triangles). Solid line: DPPC liposomes for comparison (taken from [6], reproduced with permission).

Zhai et al. [6] have used the growth temperature *T*_g_ to alter the structure of PLFE lipids. They determined the compressibilities and volume fluctuations of PLFE liposomes derived from different cell growth temperatures (*T*_*g*_ = 68, 76, and 81°C). The compressibility and volume fluctuation values of PLFE liposomes exhibit small but significant differences with *T*_*g*_.  Figure 2 shows that adiabatic compressibility (*k*_*S*_^*o*^) of PLFE liposomes changes significantly with *T*_*g*_: *k*_*S*_^*o*^(*T*_*g*_ = 68°C) > *k*_*S*_^*o*^(*T*_*g*_ = 81°C) > *k*_*S*_^*o*^(*T*_*g*_ = 76°C). For isothermal compressibility (*k*_*T*_^*o*^), isothermal compressibility coefficient (*β*_*T*_) and relative volume fluctuations, a similar, but somewhat different, trend is seen: (*T*_g_ = 68°C) > (*T*_*g*_ = 81°C) ≥ or *≈* (*T*_*g*_ = 76°C). These data indicate that, among the three employed growth temperatures, the growth temperature 76°C leads to the least compressible, and inferentially the most tightly packed PLFE lipid membranes. Note that 76°C is in the temperature range for optimal growth of *S. acidocaldarius* (75–80°C, [54, 55]). This finding suggests that membrane packing in PLFE liposomes may actually vary with the number of cyclopentane rings in a nonlinear manner, reaching maximal tightness when the tetraether lipids are derived from cells grown at the optimal growth temperatures [6].

#### 2.2.5. Future Studies of Physical Properties of Tetraether Lipid Membranes

PLFE is a mixture of GDNT- and GDGT-derived BTLs with varying numbers of cyclopentane rings. Furthermore, at any given growth temperature, there is always a broad distribution of the number of cyclopentane rings. In order to gain more insight into the effect of cyclopentane rings on compressibility and membrane volume fluctuations, it will be necessary to use purified archaeal BTLs with a well-determined number and location of cyclopentane rings. It has been reported that intact polar lipids (archaeols (diethers) and caldarchaeols (GDGT)) of the archaeon *Thermoplasma acidophilum* can be separated with single cyclopentane ring resolution by high-performance liquid chromatography (HPLC) as detected by evaporative light-scattering detection [26, 56]. However, the study by Shimada et al. on *T. acidophilum* was limited to GDGT-based BTLs. To separate intact archaea BTLs at single cyclopentane ring resolution when both GDNT- and GDGT-derived BTLs are present remains a major challenge.

**Figure 3:**
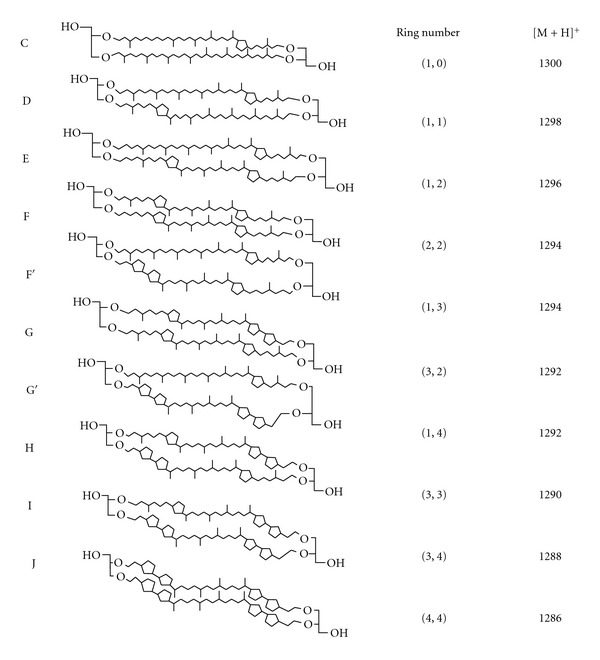
Structures of cyclopentane ring containing GDGTs previously reported to exist in archaea [7]. The number of cyclopentane rings in the first and second hydrocarbon chains is indicated in the parentheses. The mass-to-charge ratio (*m/z*) of the protonated form [M+H]^+^ for each structure is also listed.

Hydrolyzed BTLs can also be separated with single cyclopentane ring resolution using normal phase HPLC and positive ion atmospheric pressure chemical ionization mass spectrometry [7].  Figure 3 shows the structures of the cyclopentane-containing GDGT hydrophobic cores previously identified from the archaeon *Sulfolobus solfataricus*. These structures were determined by mass spectrometry. Compounds F′ and G′ (Figure 3) were reported as minor components in *S. solfataricus* [7]. The relative distribution of these GDGT structures varies from species to species. The GDGT fraction of *S. solfataricus* is dominated by those structures with one (Structures E and G, Figure 3) or two (F) biphytanyl chains with two cyclopentane rings. The distribution of GDGTs in the extract of the archaeon *M. sedula* is somewhat different. In this case, the distribution is dominated by structures containing one or two biphytanyls with one cyclopentane ring. Physical properties of liposomes made of hydrolyzed BTLs (without sugar and phosphate moieties) are not expected to be the same as those obtained from the liposomes made of intact BTLs [47].

#### 2.2.6. Disruption of PLFE Liposome Stability

While BTL liposomes (such as PLFE liposomes) exhibit remarkable stability against a number of chemical and physical stressors as mentioned above, their stability can be attenuated or abolished under certain conditions. The most striking finding in this regard is that PLFE liposomes become excessively disrupted by the presence of two archaeal proteins, namely, CdvA and ESCRT-III (ESCRT: endosomal sorting complex required for transport) [57]. CdvA is a membrane interacting protein that forms structures at mid-cell prior to nucleoid segregation. CdvA recruits ESCRT-III to membranes in order to aid in the final steps of cell division in some species of archaea. Negative stain electron microscopy revealed extensive deformation of PLFE liposomes in the presence of both CdvA and ESCRT-III together, but not individually [57]. The molecular mechanism underlying this disruption is not clear.

PLFE liposomes are “autoclavable.” However, low pH (<4) and low salt concentrations (<50 mM) are unfavorable for autoclaving PLFE-based liposomes [47]. PLFE liposomes and PLFE-based stealth liposomes (e.g., 95 mol% PLFE, 3 mol% 1,2-distearoyl-*sn*-glycerol-3-phosphoethanolamine-polyethylene glycols (2000) (DSPE-PEG(2000)) and 2 mol% DSPE-PEG(2000)-maleimide) are extraordinarily stable against autoclaving between pH 4–10 [47]. These liposomes retained their particle size and morphology against multiple autoclaving cycles. One autoclaving cycle refers to the incubation of a sample for 20 min at 121°C under a steam pressure of ~18 psi. However, at pH 2-3, one or two autoclaving cycles appeared to disrupt these liposomal membranes, causing a significant increase in particle size [47]. PLFE liposomes were more resistant to dye leakage than the gel state of conventional diester liposomes under high salt and autoclaving conditions. As the salt concentration was decreased from 160 to 40 mM, the percent of dye molecules that leaked out from PLFE-based stealth liposomes after one autoclaving cycle increased from 10.8% to 56.3% [47].

As expected, PLFE-based liposomes can also be disrupted by surfactants. The effect of the surfactant n-tetradecyl-*β*-D-maltoside (TDM) on unilamellar vesicles composed of PLFE and POPC (1-palmitoyl-2-oleoyl-sn-glycero-3-phosphocholine, a monopolar diester lipid) has been examined [58]. TDM disrupts the POPC/cholesterol vesicles effectively; however, higher concentrations (~10 times) of TDM were required to disrupt PLFE/POPC vesicles.

#### 2.2.7. Structural and Packing Properties of PLFE Monolayer Films Spread at the Interface between Air and Water

Effects of cell growth temperature, subphase temperature and pH, and lateral film pressure on PLFE lipid monolayers at the air-water interface have been examined using X-ray reflectivity (XRR) and grazing incidence X-ray diffraction (GIXD) [5]. XRR and GIXD determine the vertical and horizontal structure of the monolayers, respectively.

For PLFE derived from cells grown at 76°C, a total monolayer thickness of ~30 Å was found in the XRR measurements for all monolayers studied. This finding suggests that both head groups of a U-shaped conformation of the molecules are in contact with the subphase and that a single hydrocarbon chain region is protruded into the air. Similar U-shaped monolayer structures have been reported in other tetraether lipid membranes [59]. However, some other studies [60, 61] suggest that the U-shaped and the upright conformations may coexist in the monolayer at the same time or occur sequentially after spreading the TL lipids at the water-air interface.

At the subphase temperatures 10°C and 20°C, large, highly crystalline domains were observed by GIXD; and the thickness of the crystalline part of the monolayer is slightly larger than 30 Å, which indicates a tight packing of the whole lipid monolayer, including both the hydrocarbon chain and the head group regions. The area per hydrocarbon chain of PLFE (~19.3 Å^2^) found by GIXD is significantly smaller than that of DPPC (~23.2 Å^2^) or 1,2-dipalmitoyl-*sn*-glycero-3-phosphoglycerol (DPPG) (~22.6 Å^2^). In fact, both the two hydrocarbon chains of a single PLFE lipid and the chains of neighbouring lipid molecules adopted an extremely tight packing.

For PLFE lipids derived from cells grown at higher temperatures, a slightly more rigid structure in the lipid dibiphytanyl chains was observed. However, the growth temperature, inferentially the number of cyclopentane rings, does not affect the parameters of the unit cell in GIXD measurements. This suggests that there exists a nearly identical crystalline packing of all the PLFE lipids examined and that, at high film pressures, membrane packing is primarily governed by the lipid headgroup region [5]. It is interesting to mention that the lack of cyclopentane rings in the bipolar tetraether lipids from *M. hungatei* has been suggested to be the cause of the U-shaped configuration adopted by these lipids in the monolayer film at the air-water interface [62]. Apparently, the presence of cyclopentane rings would hinder the dibiphytanyl hydrocarbon chains from bending to form the U-shaped configuration.

## 3. Physical Properties of Membranes Made of Synthetic Tetraether Lipids

The process of isolating well-defined archaeal tetraether lipids can be difficult and time consuming. In addition, archaeal tetraether lipids have several structural features distinctly different from conventional diester lipids. Therefore, it is rather difficult to elucidate the structure-activity relationship for each of the individual structural features when using native archaeal lipids. To resolve these problems to some extent, synthetic tetraether lipid analogues have been used [63–67].

### 3.1. Importance of the Stereochemistry of the Cyclopentane Ring

**Figure 4:**
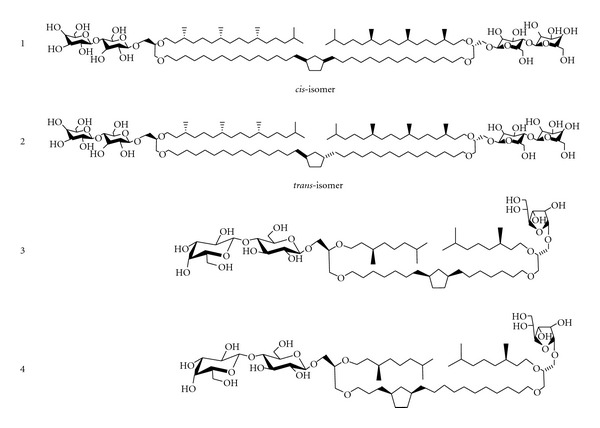
Synthetic tetraether lipids that have been used to study the effect of configuration (Compounds 1 and 2 [8, 9]) and position (Compounds 3 and 4 [10]) of the cyclopentane rings on membrane properties.

Jacquemet et al. were able to study the effect of the stereochemistry of the cyclopentane ring on BTL membrane properties by using two synthetic tetraether lipids [8, 9] (Compounds 1 and 2 in Figure 4). Both lipids have a bridging hydrocarbon chain with a single 1,3-disubstituted cyclopentane ring at the center. The substitutes on the ring are ether-linked to C3 of the two opposite glycerol moieties, while C2 of the glycerols is ether-linked to a phytanyl chain and C1 is linked to a lactosyl polar headgroup (Figure 4). The only difference between these two isomers is the configuration (*cis or trans*) of the 1,3-disubstituted cyclopentane ring [8, 9].

The *trans-*isoform showed multilamellar vesicles whereas the *cis-*counterpart led to nonspherical nanoparticles, as revealed by cryo-transmission electron microscopy [8]. Small angle X-ray scattering (SAXS) studies further showed that the *cis-*isomer exhibited L_c_-L_*α*_-Q_II_ (cystal, lamellar, and bicontinuous cubic phase (Pn3 m), resp.) phase transitions whereas the *trans-*isomer remained in L_*α*_ phase from 20 to 100°C. The electron density profiles calculated from the SAXS data were consistent with a stretched conformation of these synthetic BTLs within the L_*α*_ phase [9]. The difference in the phase behaviors was attributed to the conformation equilibrium of 1,3-disubstituted cyclopenatne rings. The dominant conformational motion in cyclopentane is pseudorotational [68]. Pseudorotation is more restricted for the *trans-*isomer whereas several more orientations of the two substituents on the ring can be created for the *cis*-1,3-dialkyl cyclopentane ring [9, 68, 69]. Even though this study shows that the stereochemistry at the cyclopentane ring has a dramatic influence on membrane properties, more work is still required in order to explain why liposomes made of PLFE, which naturally occurs and contains *trans*-1,3-disubstituted cyclopentyl rings, can undergo the L_*α*_-to-Q_II_ phase transition [48, 49], while the synthetic *trans* BTL (Figure 4) cannot [9]. Note that the placement and the number of cyclopentane rings are different between PLFE lipids (Figure 1) and the synthetic BTLs mentioned above (Figure 4). Apparently, BTLs with subtle differences in chemical structures can display distinctly different phase behaviors.

The difference in the polar headgroups between PLFE and the above-mentioned synthetic BTLs also leads to other subtle structural differences. The *d*-spacing of PLFE liposomes increases with increasing temperature [48], which is contrary to that obtained from the synthetic *trans-*isomer mentioned above (Compound 2 in Figure 4) [9]. The increased *d*-spacing with temperature is probably due to an increase in hydration at the polar headgroup of PLFE [48]. For unknown reasons, there is no change in hydration at the polar (lactosyl) headgroups in those two synthetic stereo-isomers [9].

### 3.2. Influence of the Position of the Cyclopentane Ring

Brard et al. studied the effect of the position of the cyclopentane ring on physical properties of tetraether lipid membranes [66]. They synthesized two tetraether glycolipids, each of which contains a single *cis*-1,3-disubstituted cyclopentane ring in the bridging chain. One glycolipid contained a cyclopentane ring in the middle of the bridging chain while the other had one at three methylene units from the glycerol backbone (Compound 3 and 4 in Figure 4). This helped them determine the influences of the different positions of the cyclopentane ring.

The cyclopentane ring position appears to have a profound impact on hydration properties, lyotropic liquid crystalline behavior, and membrane organization [66]. Moreover, the synthetic BTL with the cyclopentane ring positioned at the center (Compound 3 in Figure 4) can be completely dispersed in water, and it can form sponge-like structures as revealed by electron microscopy. In contrast, the compound with the cyclopentane ring away from the center (Compound 4 in Figure 4) can only be partitially dispersed in water and it forms multilamellar vesicles. It has been suggested that the position of the cyclopentane ring in the bridging chain influences the orientation of the glycosidic polar headgroups attached to the glycerol backbone, which leads to different membrane organizations [66].

## 4. Membrane Properties Revealed by Computer Simulations

### 4.1. Effect of Cyclopentane Rings on Membrane Packing and Headgroup Orientation

An increase in growth temperature is known to increase the number of cyclopentane rings in the dibiphytanyl chains of archaeal lipids [23]. The number of cyclopentane rings may vary from 0 to 4 in each biphytanyl chain (i.e., 0 to 8 per dibiphytanyl unit). To evaluate how the number of cyclopentane rings might affect membrane packing, Gabriel and Chong have conducted molecular modeling studies on a membrane containing 4 × 4 GDNT molecules (with sugar moieties, Figure 1) [27]. It was found that when 8 cyclopentane rings are contained, the headgroup of GDNT runs almost parallel to the membrane surface. However, without containing any rings, the headgroup is oriented perpendicular to the membrane surface. The molecular modeling further showed that an increase in the number of cyclopentane rings in the dibiphytanyl chains of GDNT from 0 to 8 made GDNT membrane packing tighter, more rigid, and more negative in interaction energy (−156.5 kcal/mol for 0 cyclopentane ring to −191.6 kcal/mol with 8 rings [27]). The resulting energy lowering effect is neither due to the decrease in polar headgroup separation, nor the change in the van der Waals interactions. Instead, it is due to the more favorable hydrogen bonding, and bonded interactions including harmonic bending, theta expansion bond angle bending, dihedral angle torsion, and inversion [27].

### 4.2. Effect of Macrocyclic Linkage on Membrane Properties

Most archaeal BTLs are macrocyclic molecules with two biphytanyl hydrocarbon chains linked to two opposite glycerol or calditol backbones (illustrated in Figure 1 for the case of PLFE). The effect of the macrocyclic linkage on membrane properties has been studied by molecular dynamics simulations [10, 70, 71]. For simplicity, coarse graining approaches were employed and BTL molecules were modeled as di-monopolar lipids such as di-DPPC [10] and diphytanyl phosphatidylcholine (DPhPC) [70]. In essence, two monopolar molecules were tethered together either at one pair of the hydrocarbon chains (acyclic di-DPPC or di-DPhPC) or at both pairs (cyclic di-DPPC or di-DPhPC). The simulations showed that in the membranes composed of macrocyclic BTL-like molecules, the upright configuration gains favor over the U-shaped configuration [70]. The macrocyclic linkage also leads to a condensing effect on the membrane surface, increases the order of the lipid hydrocarbon chains, slows lateral mobility in the membrane, and increases membrane thickness [10, 70, 71]. Furthermore, the molecular dynamics simulations made by the dissipative particle dynamics method [71] revealed the formation of two types of membrane pores. Hydrophobic pores are unstable and transient and exist at the low temperature. Hydrophilic pores are more stable with much longer lifetimes and are observed at high temperatures. The simulation data [71] suggested that hydrophilic pores can lead to the rupture of membrane vesicles. More intriguingly, it was proposed that hydrophobic pores, which occur at low temperatures, may result in the permeation of encapsulated small molecules [71]. This implies that although BTL membranes are extremely stable and tightly packed, some small leakage of entrapped molecules can still occur due to either the formation of hydrophobic pores [71] or membrane volume fluctuations [6, 50] (discussed earlier).

## 5. Applications of Tetraether Lipid Membranes

The extraordinary stability of tetraether lipid membranes against a variety of physical and biochemical stressors has provided the basis for using these lipids to develop technological applications. BTLs can be used as a stable lipid matrix for biosensors [72], a light harvesting device [73], and nanoparticles for targeted imaging and therapy (reviewed in [12, 74]).

It has been proposed that liposomes made of archaeal lipids (also called archaeosomes) are taken up via a phagocytosis receptor in the plasma membrane of the target cells [75]. This uptake occurs in a liposomal composition-dependent manner [75]. Total polar lipids from the archaeon, *Halobacterium salinarum CECT 396*, have been used to make archaeosomes and archaeosomal hydrogels as a possible topical delivery system for antioxidants [76]. Compared to conventional liposomes, those archaeosomes and archaeosomal hydrogels showed better stability and more sustained drug release [76]. It is of interest to extend their study from diethers (abundant in *Halobacterium salinarum CECT 396*) to tetraether lipids (e.g., PLFE lipids isolated from thermoacidophiles). BTL-based liposomes are suitable for oral delivery of therapeutic agents because BTL liposomes are stable against the harsh conditions (such as bile salts, pancreatic enzymes, and low pH) in the gastrointestinal tract [77]. Tetraether lipid membranes have also been tailored and evaluated as an intranasal peptide delivery vehicle [78]. PEGylated tetraether lipids have been synthesized and tested for their stability in test tubes and for liposomal encapsulation potential [79]. Knowledge gained from the physical studies of cyclopentane rings, sugar moieties, and macrocyclic structures should help to optimize the numerous potential applications.

## Abbreviations

AIP: archaetidylinositol phosphate
BTL: bipolar tetraether lipids
DMPC: 1,2-dimyristoyl-*sn*-glycero-3-phosphocholine
DPhPC: 1,2-di-O-phytanyl-*sn*-glycero-3-phosphocholine
DPPC: 1,2-dipalmitoyl-*sn*-glycero-3-phosphocholine
DPPG: 1,2-dipalmitoyl-*sn*-glycero-3-phosphoglycerol
DSC: differential scanning calorimetry
DSPE-PEG: 1,2-distearoyl-*sn*-glycerol-3-phosphoethanolamine-polyethylene glycols
ESCRT: endosomal sorting complex required for transport
GDGT: caldarchaeol
GDNT: calditoglycerocaldarchaeol
GIXD: grazing incidence X-ray diffraction
HPLC: high-performance liquid chromatography
PLFE: polar lipid fraction E
POPC: 1-palmitoyl-2-oleoyl-*sn*-glycero-3-phosphocholine
PPC: pressure perturbation calorimetry
TDM: n-tetradecyl-*β*-D-maltoside
TL: tetraether lipids
TPL: total polar lipids
XRR: X-ray reflectivity.
